# 

**Published:** 1910-10

**Authors:** 


PUBLISHED MONTHLY. SUBSCRIPTION $1.00

### ATLANTA JOURNAL-RECORD OF MEDICINE

Successor to { Atlanta Medical and Surgical Journal, Established 1855. and Southern Medical Record, Established 1870. } 56th Year

Vol. LVI. ATLANTA, GA., OCTOBER, 1910. No. 7

